# Supplementary material for: Integrative multiomic analyses of dorsal root ganglia in diabetic neuropathic pain using proteomics, phospho-proteomics, and metabolomics
Source: Sci Rep. 2022 Oct 11;12:17012. doi: 10.1038/s41598-022-21394-y (PMC9553906; doi:10.1038/s41598-022-21394-y)
Supplement: Supplementary file 2 — Supplementary Information. [file 41598_2022_21394_MOESM2_ESM.docx]

# **Integrative Multiomic Analyses of Dorsal Root Ganglia in Diabetic Neuropathic Pain using Proteomics, Phospho-proteomics, and Metabolomics**

Megan Doty^1^, Sijung Yun^2^, Yan Wang^3^, Minghan Hu^1^, Margaret Cassidy^1^, Bradford Hall^1^, Ashok B. Kulkarni^1^

^1^Funtional Genomics Section, National Institute of Dental and Craniofacial Research, National Institutes of Health, Bethesda, MD 20892.

^2^Predictiv Care, Inc, Mountain View, CA 94040.

^3^Mass Spectrometry Facility, National Institute of Dental and Craniofacial Research, National Institutes of Health, Bethesda, MD20892

**Supplemental Data**

**Supplementary Figure 1**

Plotted results of PCA performed with metabolomic data. DRG donor condition is indicated by color and tissue storage method (snap frozen (SF) or RNAlater (RNA)) is indicated by shape of the point.

**Supplementary Figure 2**

Concentration for amino acids in the DRG as detected using quantitative mass spectrometry panels. Amino acids are arranged according to highest average concentration in control human DRG tissues. CON indicates control, DPN indicates Diabetic Peripheral Neuropathy. Amino acids labeled with standard 3 letter abbreviations.

**Supplementary Figure 3**

Plotted results of (A) PCA and (B) t-SNE performed with proteomic data. For both plots, DRG donor condition is indicated by color and donor sex (female (F) or male (M)) is indicated by shape of the point.

**Supplementary Figure 4**

Plotted results of (A) PCA and (B) t-SNE performed with phospho-proteomic data. For both plots, DRG donor condition is indicated by color and donor sex (female (F) or male (M)) is indicated by shape of the point.

**Supplementary Figure 5**

A) Specificity of TNR antibody was confirmed using positive (mouse brain) and negative (mouse liver and spleen) controls. B) and C) are images from the same membrane, the membrane was cut to avoid stripping. B) displays TNR labeling. C) displays alpha-tubulin labeling. All images were acquired using auto-exposure setting on the FlourChem M imager.

**Supplementary Figure 6**

Specificity of p-eEF2 (A) and eEF2 (B) antibodies was confirmed using positive a control (mouse brain). P-eEF2 antibody reacts with a truncated p-eEF2 product. Images C), D), and E) are from the same membrane, the membrane was cut to avoid stripping when possible. C) and D) show the same higher molecular weight piece of the membrane. C) displays p-eEF2 labeling. D) displays eEF2 labeling. E) displays alpha-tubulin labeling on a lower molecular weight piece of the membrane. All images were acquired using auto-exposure setting on the FlourChem M imager.

**Supplementary Figure 7**

Full membranes for 2D gels, labeled for SPP1 show molecular weight and pI of SPP1 in DPN and control samples. Location of a loading control was determined by overlaying developed images here with a Coomassie blue stained image of the same membrane (tropomyosin, pI = 5.2) is indicated with an asterisk (*). Images were acquired with an Amersham Imager 600 with 1 minute exposure.

**Supplementary Table 1**

Metabolites identified as altered with DPN. Ratios in relative abundance data are recorded as DPN/control. For metabolites for which identity and abundance were validated with a quantitative panel, p-values were calculated with quantitative data, results of this are included on the far right.

**Supplementary Table 2**

Proteins identified as significantly altered with DPN (p-value adjusted for covariates < 0.05). Ratios are recorded as DPN/control.

**Supplementary Table 3**

Phospho-peptides identified as significantly altered with DPN (p-value adjusted for covariates < 0.05). Each row indicates a phospho-peptide with the phosphorylation site noted in the column labeled “p-site”. The abundance ratio of the indicated peptide’s master protein is noted in the column labeled “Master protein ratio”. Ratios are recorded as DPN/control.

**
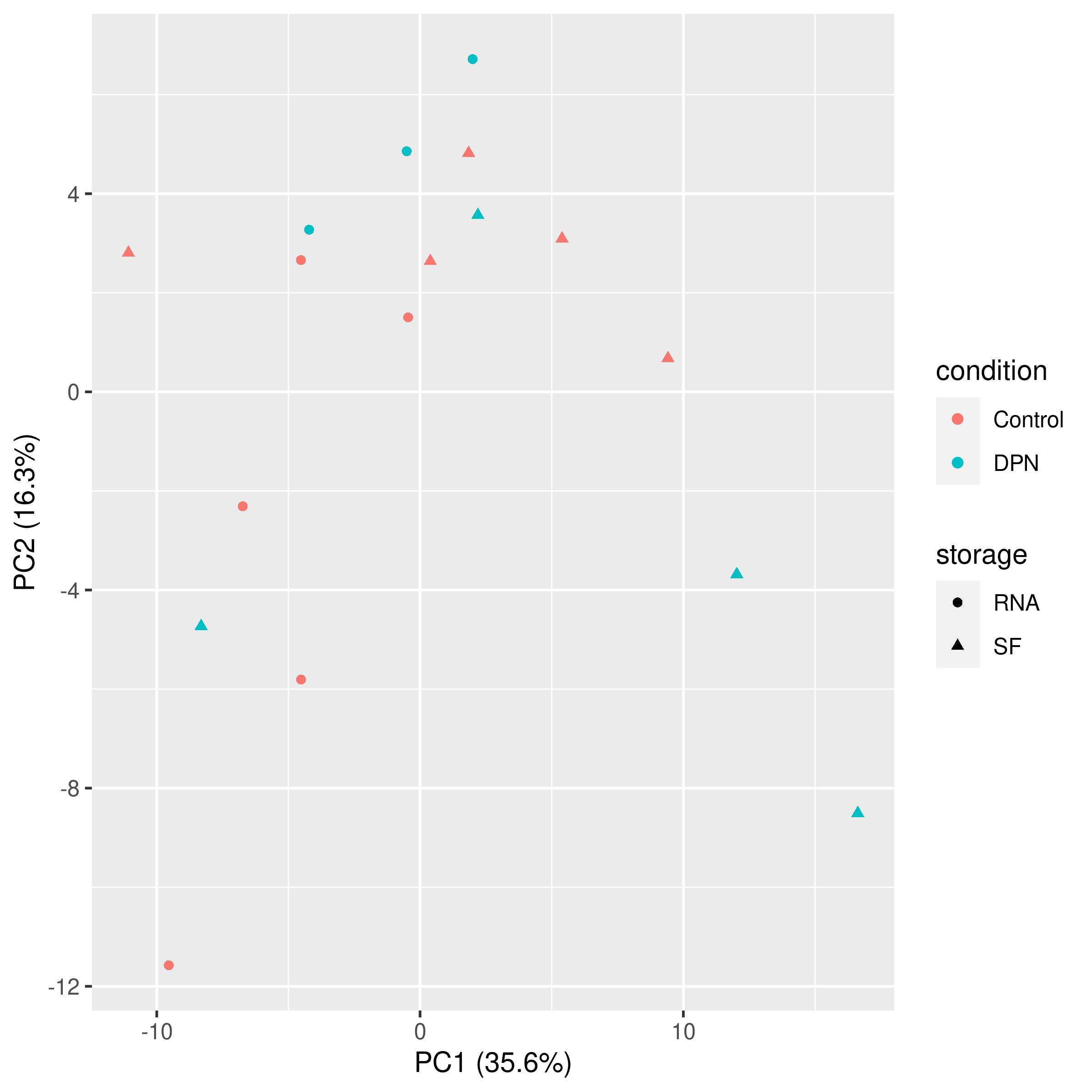
**

**Supplementary Figure 1**

Plotted results of PCA performed with metabolomic data. DRG donor condition is indicated by color and tissue storage method (snap frozen (SF) or RNAlater (RNA)) is indicated by shape of the point.

**
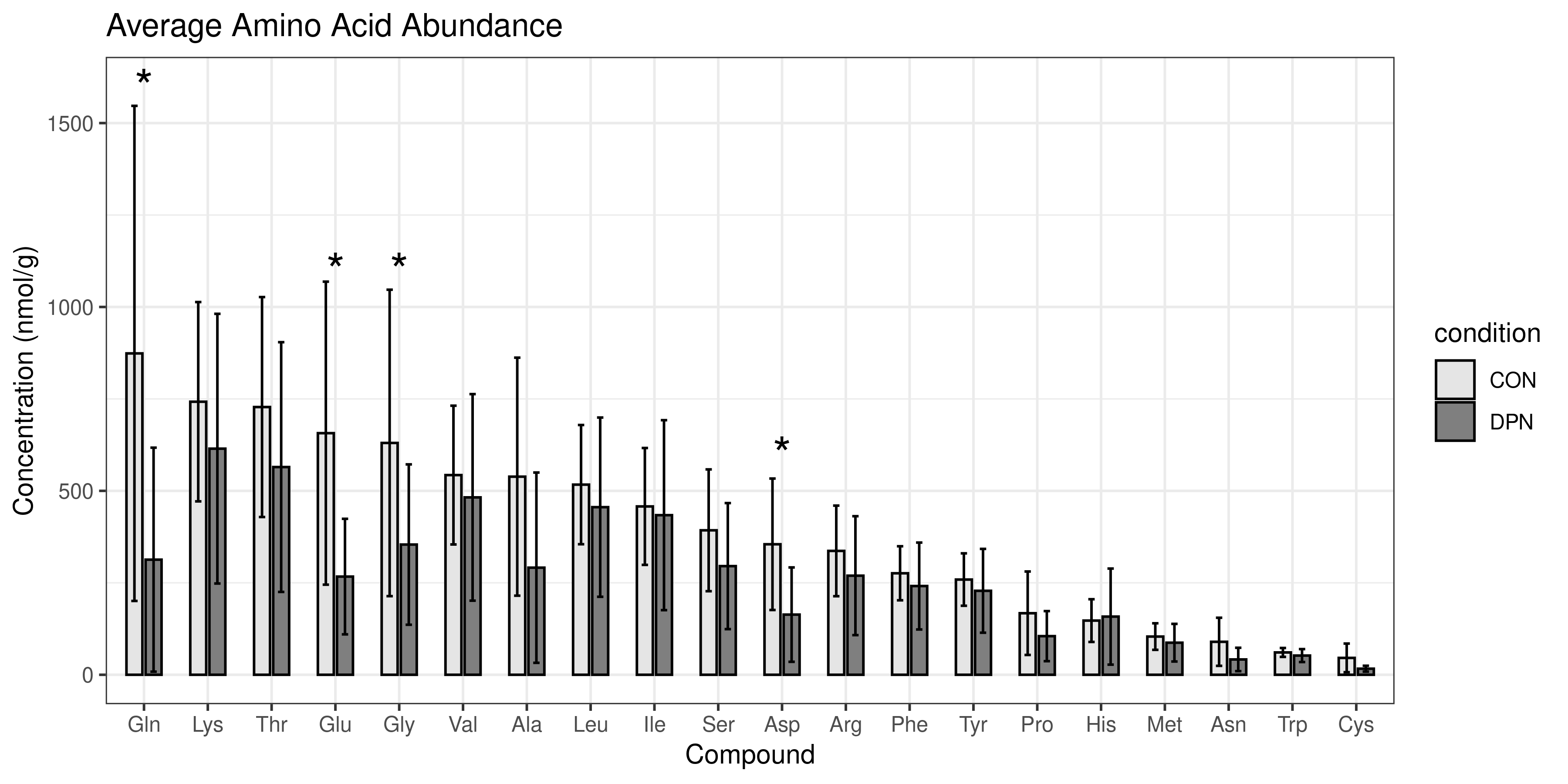
**

**Supplementary Figure 2**

Concentration for amino acids in the DRG as detected using quantitative mass spectrometry panels. Amino acids are arranged according to highest average concentration in control human DRG tissues. CON indicates control, DPN indicates Diabetic Peripheral Neuropathy. Amino acids labeled with standard 3 letter abbreviations.

**
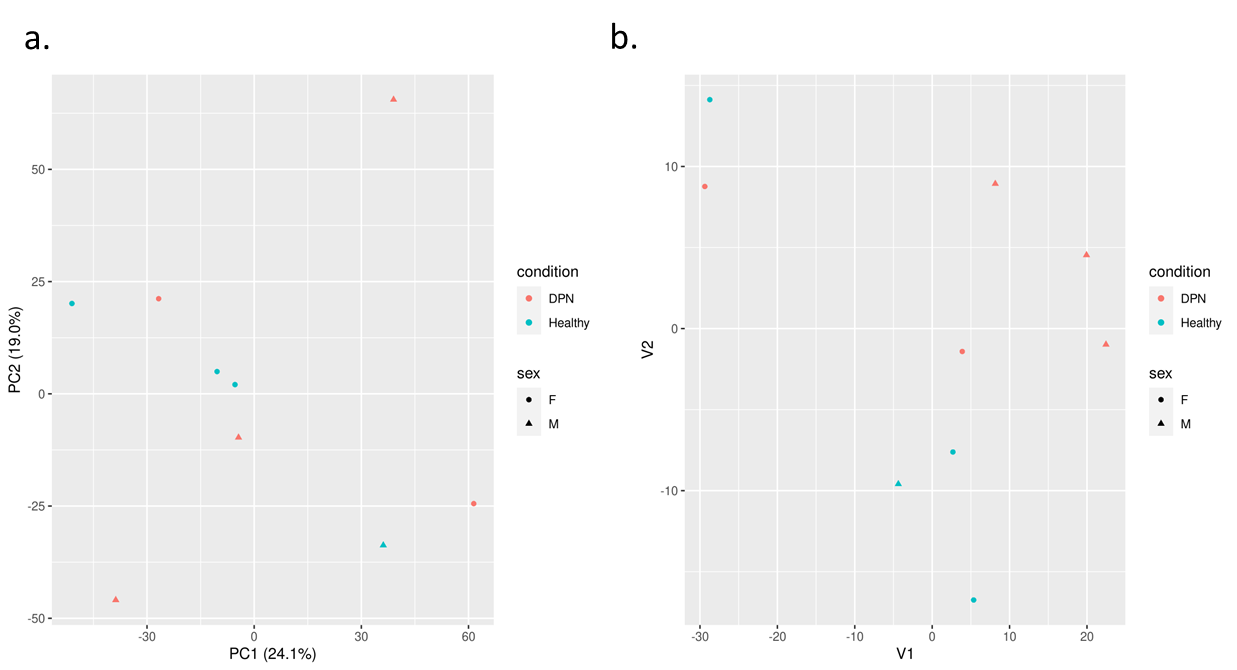
**

**Supplementary Figure 3**

Plotted results of (A) PCA and (B) t-SNE performed with proteomic data. For both plots, DRG donor condition is indicated by color and donor sex (female (F) or male (M)) is indicated by shape of the point.

**
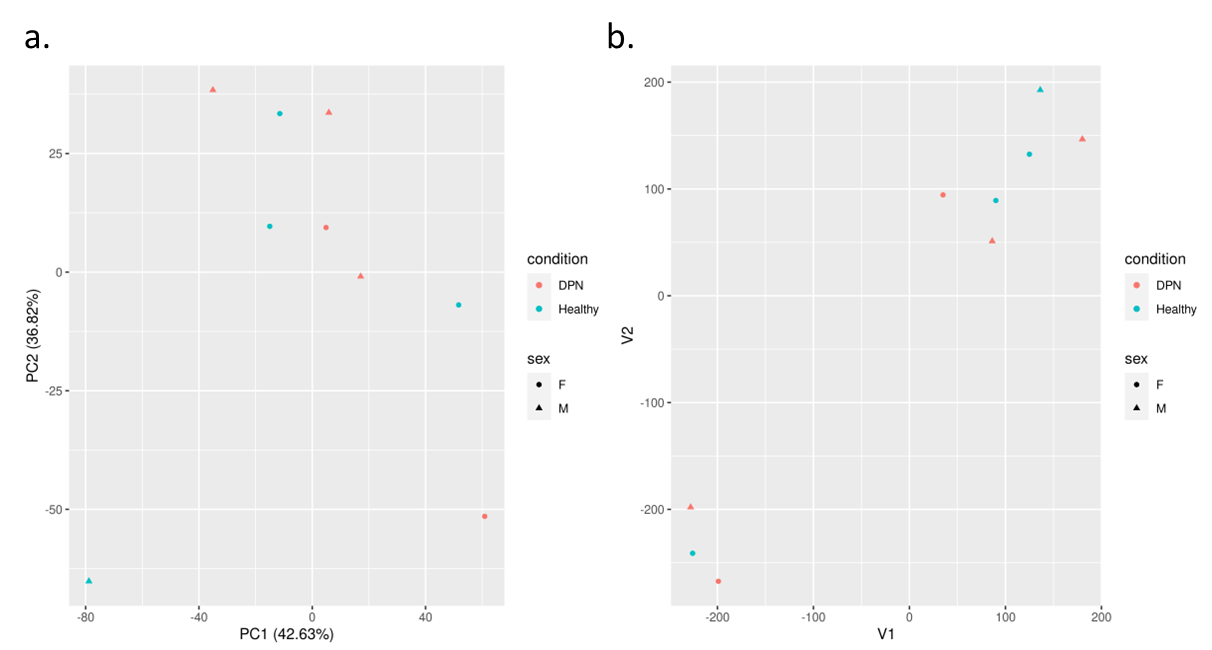
**

**Supplementary Figure 4**

Plotted results of (A) PCA and (B) t-SNE performed with phosphor-proteomic data. For both plots, DRG donor condition is indicated by color and donor sex (female (F) or male (M)) is indicated by shape of the point.

**
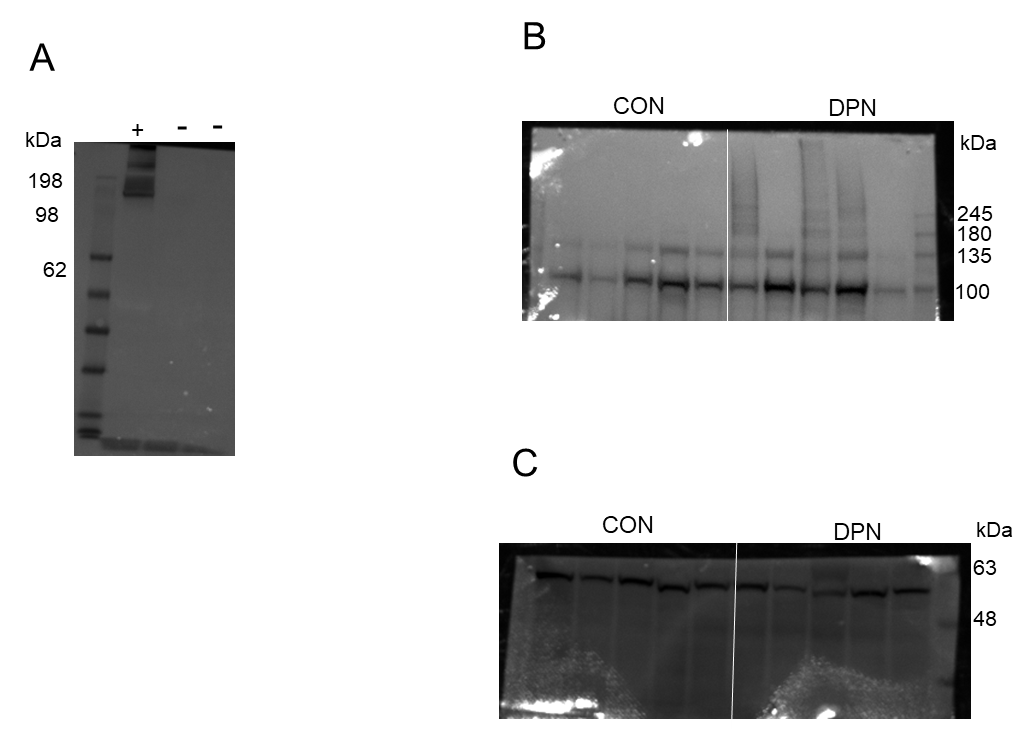
**

**Supplementary Figure 5**

A) Specificity of TNR antibody was confirmed using positive (mouse brain) and negative (mouse liver and spleen) controls. B) and C) are images from the same membrane, the membrane was cut to avoid stripping. B) displays TNR labeling. C) displays alpha-tubulin labeling. All images were acquired using auto-exposure setting on the FlourChem M imager.

**
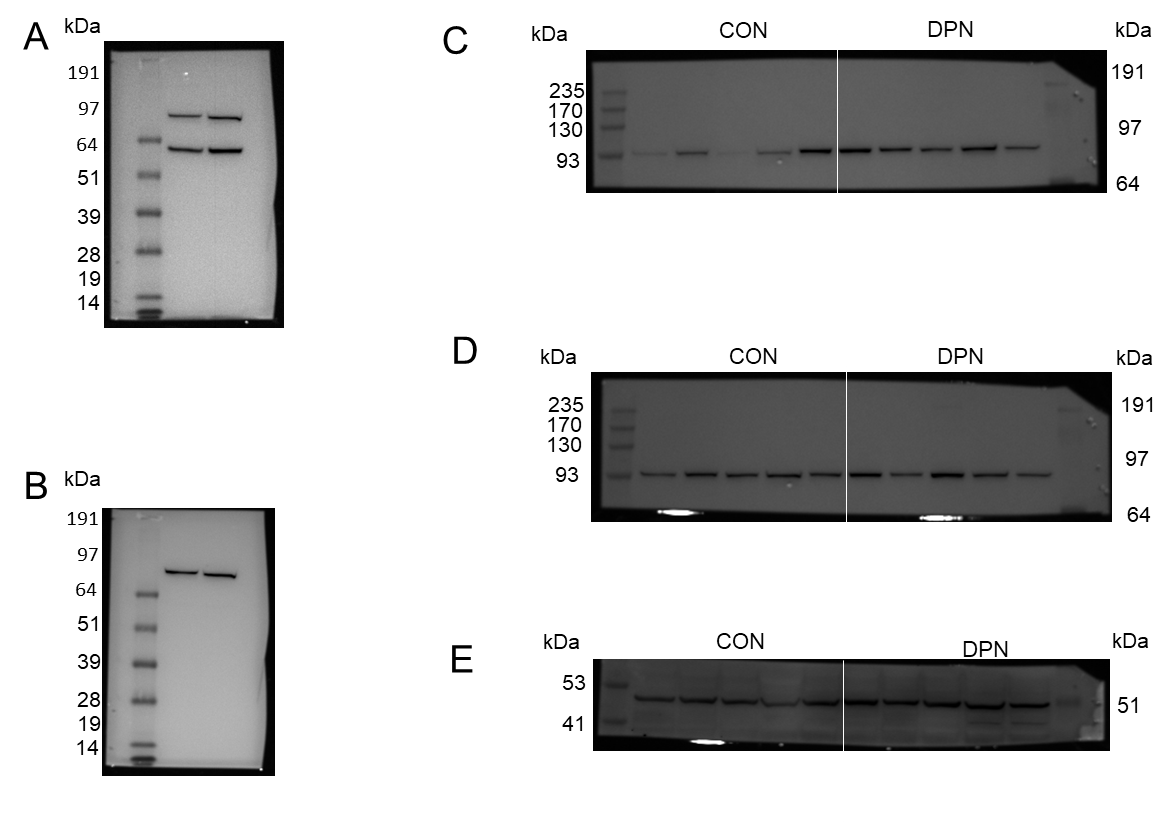
**

**Supplementary Figure 6**

Specificity of p-eEF2 (A) and eEF2 (B) antibodies was confirmed using positive a control (mouse brain). P-eEF2 antibody reacts with a truncated p-eEF2 product. Images C), D), and E) are from the same membrane, the membrane was cut to avoid stripping when possible. C) and D) show the same higher molecular weight piece of the membrane. C) displays p-eEF2 labeling. D) displays eEF2 labeling. E) displays alpha-tubulin labeling on a lower molecular weight piece of the membrane. All images were acquired using auto-exposure setting on the FlourChem M imager.


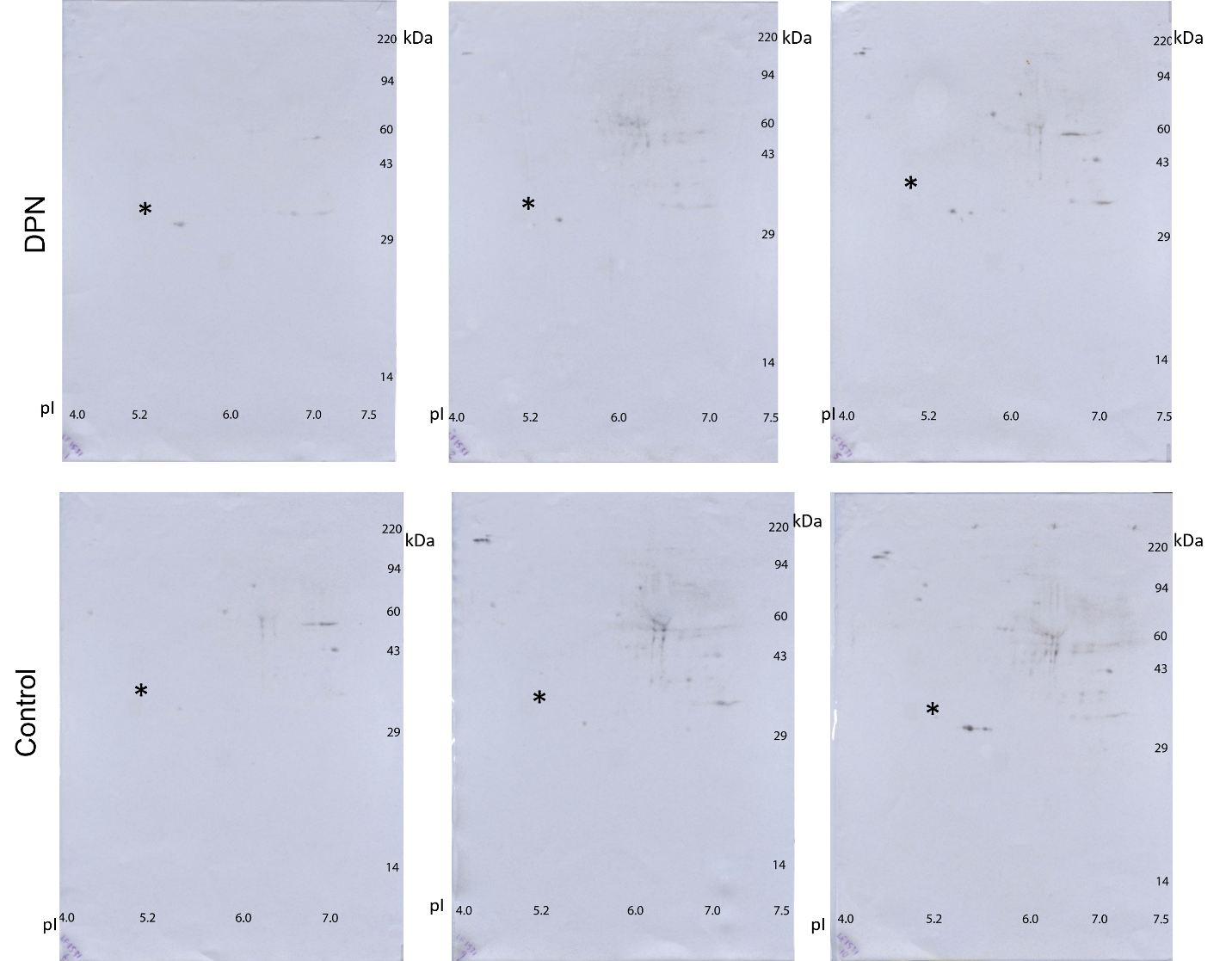


**Supplementary Figure 7**

Full membranes for 2D gels, labeled for SPP1 show molecular weight and pI of SPP1 in DPN and control samples. Location of a loading control was determined by overlaying developed images here with a Coomassie blue stained image of the same membrane (tropomyosin, pI = 5.2) is indicated with an asterisk (*). Images were acquired with an Amersham Imager 600 with 1 minute exposure.
